# Institutional Needs

**Published:** 1915-12-04

**Authors:** 


### Institutional Needs.

### COOKING APPARATUS.

CARRON COMPANY, FALKIRK.

THE Carron Company of Falkirk are manufacturers of cooking apparatus of every conceivable kind—for coal, gas, coke, steam, and electricity—on a very extensive scale, and institutional authorities who contemplate installing new appliances of this nature would be well advised to study the profusely illustrated catalogues issued by this company. The company are prepared to specify and quote for any style, size, or combination of cooking apparatus, from an ordinary range to the most elaborate installation, and are always pleased to suggest and submit drawings and estimates free of charge. Their apparatus is installed in a considerable number of hospitals and infirmaries.

